# Practice makes the expert: The importance of training volunteers in the generation of phenological data from photographs of biodiversity observation platforms

**DOI:** 10.1371/journal.pone.0282750

**Published:** 2023-03-07

**Authors:** Julieta Salomé-Díaz, Jordan Golubov, Omar Díaz-Segura, Ma. Cristina Ramírez-Gutiérrez, Sarah Sifuentes de la Torre, Patricia Koleff, Esther Quintero, Armando Jesús Martínez

**Affiliations:** 1 Instituto de Ecología, Universidad Nacional Autónoma de México, Ciudad Universitaria, Coyoacán, Ciudad de México, México; 2 Posgrado en Ciencias Biológicas, Unidad de Posgrado, Edificio A, 1° Piso, Circuito de Posgrados, Ciudad Universitaria, Coyoacán, Distrito Federal, México; 3 Laboratorio de Taxonomía y Sistemática Vegetal, Universidad Autónoma Metropolitana, Coyoacán, Ciudad de México, México; 4 Facultad de Ciencias, Universidad Nacional Autónoma de México, Ciudad Universitaria, Coyoacán, Ciudad de México, México; 5 Comisión Nacional para el Conocimiento y Uso de la Biodiversidad, Tlalpan, Ciudad de México, Mexico; 6 Instituto de Neuroetología, Universidad Veracruzana, Dr. Luis Castelazo s/n, Col. Industrial Animas, Xalapa, Veracruz, México; INRA/Sorbonne University, FRANCE

## Abstract

Phenology studies the time at which events in the life cycle of a species occur sand how they are related to environmental cues. Patterns of change in phenology at different scales can be used as an indicator of ecosystem changes and climate change, but the data necessary to detect these changes can be difficult to obtain due to their temporal and regional dimensions. Citizen science can contribute to generate large amounts of data on phenological changes at wide geographical scales that would be almost impossible for professional scientists to generate, but the quality and reliability of these data are often questioned. The objective of this study was to evaluate the use of a biodiversity observation citizen science platform based on photographic information as a potential source of large-scale phenological information, and to identify the key benefits and limitations of this type of information source. We used the Naturalista photographic databases for two invasive species in a tropical region: *Leonotis nepetifolia* and *Nicotiana glauca*. The photographs were classified into different phenophases (initial growth, immature flower, mature flower, dry fruit) by three groups of volunteers: a group of experts, a trained group with information on the biology and phenology of both species, and an untrained group. The degree of reliability of the phenological classifications was estimated for each group of volunteers and each phenophase. The degree of reliability of the phenological classification of the untrained group was generally very low for all phenophases. The group of trained volunteers showed accuracy levels for the reproductive phenophases that equaled the degree of reliability among the expert group, regardless of species, and was consistent across phenophases. We conclude that volunteer classification of photographic information contained in biodiversity observation platforms can provide phenological information with high geographic coverage and an increasing temporal coverage on general phenological patterns of species with wide distributions but has limited applicability in the identification of exact start and end dates. and peaks of the different phenophases.

## Introduction

### Phenology as a science and its application

Phenological responses have a genetic component such that there is an ecological fit between plant development and environmental conditions. These external cues cause phenophases of coexisting individuals to respond more or less synchronously to environmental conditions [1–3]. The close environment-development response has allowed for the use of phenological data to describe the synchronization of ecological interactions [4], determine the structure of plant communities [5,6], assess the dynamics of nutrient uptake and CO_2_ levels and other gases at the ecosystem level [7,8], and optimize agricultural and horticultural strategies [9–11], among others. In addition, the sensitivity of some phenophases to environmental temperature has made phenology an effective tool for monitoring the impacts of global climate change (GCC) on plants and animals [12]. For example, changes in growing season patterns [13], as well as shifts in flowering and fruit production [14] in recent decades have been detected and correlated with environmental changes attributed to GCC. These changes have in turn had repercussions on ecological components [4], agricultural practices [11,15,16], and ecosystem goods and services [17–19]. Nonetheless, using phenological at a regional or global scale involves large amounts of quantitative and standardized data on phenological attributes coming from a wide spatial and temporal window, as well as time series of an established association between phenological response and environmental variables, usually accessible by national meteorological services.

Many phenological studies are based on measurements from a sample of individuals within a population or community [20]. This type of research generates detailed and precise data on the timing and intensity of phenophases and their association with environmental requirements in a population or a small geographic area, but intrinsic limitations in terms of number of species, selected methodology, and the geographic and temporal dimensions significantly restrict or make impossible potential extrapolation of phenological response patterns to environmental variations throughout an entire distribution range [7,20,21].

In order to identify patterns of phenological change at regional or global scales, or assess phenological changes in species with a wide spatial distribution and environmental tolerance, phenological monitoring can be carried out using lower resolution but high coverage data as input. That is, using large amounts of data from a wide geographical range, over several years, and not restricted by complex experimental designs nor controlled or monitored micro-environmental variables. For example, components from the phenology of communities or ecosystems can be measured using remote sensing data (*e*.*g*. aerial photographs or satellite images [22], and the phenology at regional or higher scales of particular species that cannot be studied using remote sensing methods, could be studies using herbarium records, or through collaborations with networks of non-expert volunteers who make nature observations or monitor the phenological stages of plants and animals at national or regional levels [20,23,24].

### Citizen science as an input for phenological studies

Although there is no single definition of citizen science, this term is used to refer to projects that include the participation of non-expert volunteers in the generation and/or analysis of information that contributes to scientific progress [25,26]. This activity allows gathering and analyzing data with a broad temporal and geographic scope, which would otherwise be impossible or cost restrictive. For this reason, data and sampling citizen science protocols have been to study bird diversity patterns [27], abundance and distribution of tree species [28], assessment of anthropogenic impact on natural communities [29], environmental monitoring [30], and even for the classification of stars [31]. Phenological studies have used citizen data through the National Phenology Network (NPN) portal (https://www.usanpn.org/), a platform that brings together phenological data recorded by both citizen scientists and experts, some of which has led to peer-reviewed publications ranging from phenological descriptions of a species [32] to proposals for the control of invasive species [33], and models of phenological response to climate change scenarios [34] (see https://www.usanpn.org/publications for a list of articles generated using NPN data).

Although phenological data are one of the indicators used in Global Climate Change (GCC) assessments, most countries do not have a unified platform to gather phenological information. The NPN-USA is one of the few platforms that collects, stores, and shares national-level phenological information for more than 1,300 species, including data on presence/absence and intensity of different phenophases [35]. Since 2007, this platform has established a general monitoring approach to phenology, with monitoring protocols for plants, insects, fish, amphibians and reptiles, and birds and mammals. The plant protocol distinguishes 24 phenophases, which may or may not be present in any of the 11 groups in which these organisms are classified [35]. For the recording of observations, the system provides standardized formats in which the presence/absence of each phenophase and the number of structures of interest in each phenophase (for example, from 3 to 10 flowers) or the proportion of the individual that presents each phenophase of interest (for example, 5–24% of the plant with leaves changing color) is recorded, but it does not require the registration of visual evidence such as photographs. To increase the quality of the data obtained, the program has informative and illustrated material on botanical principles and definition of phenophases [35]. In Europe, the Pan-European Phenology Project (PEP725) brings together and standardizes phenological data that have been collected by European phenology networks over the years and makes them freely available in a single unified database. In this project, each collaborating phenology network collects data (through volunteers such as PhenoWatch and the Swedish National Phenology Network) under established guidelines, which are then curated by a PEP725 committee. This project contains data from more than six decades, 265 species and 46 phenophases that follow the BBCH scale [36]. In India, the SeasonWatch citizen science project has promoted the phenological monitoring of eight vegetative and reproductive phenophases of more than 130 common Indian species since 2010. This project allows users to record, on a weekly basis, the presence and abundance of leaves, flowers, and fruits in different phenological stages, with the help of illustrated resources (https://www.seasonwatch.in).

Apart from the citizen science networks designed to gather phenological data, scientists have access to other sources to extract phenological information. Biodiversity observation platforms such as iNaturalist and the associated partners sites in different countries, such as Naturalista in Mexico, include photographic documentation with a geolocation, which helps relate geographic and temporal data and presence of species. This data has contributed to the generation of animal and plant species distributions [37–40], but the derived information as an input for phenological information has rarely been assessed [but see 25,42]. Although this platform has the option of recording phenological annotations for observations, that is, indicating the phenophase of the registered organism, these annotations are rarely made. For example, in Naturalista, which is the collaborative platform between the National Commission for the Knowledge and Use of Biodiversity (CONABIO) in Mexico and iNaturalist, few records for Mexico have phenological annotations (data obtained from naturalista.mx), perhaps due to a lack of obligation to record this information, or lack of interest or knowledge about phenological recording. Although these platforms are not explicitly aimed at generating data on phenology, by including photographs they provide a very useful visual verification resource for researchers that allows for since it allows corroboration of the species identity, the phenophases that occur, and the state of cultivation, which is often a problem with citizen science observations without photographic records [24,41].

The aim of this study was to use Naturalista, a biodiversity observation platform with photographic resources, as an input of phenological information at a national scale in Mexico to evaluate the benefits and limitations of this information source in the generation of phenological information in a wide geographical area. Since some of these platforms are not specifically designed to capture phenological data, one of the specific goals was to propose a methodology to reliably obtain and classify phenological information from photographic records of species presence, with the participation of citizen scientists. We assessed whether the quality of phenological classification by volunteers or citizen scientists changed after a brief but concise training on basic biology and phenology, and compared the level of agreement in phenological classification among groups of expert scientists, non-expert volunteers with training, and non-expert volunteers without training.

## Methods

This study was conducted using three components: (1) a photographic database of the Naturalista digital platform (https://www.naturalista.mx/), where photographs were classified according to the phenological phases they represented; (2) a group of five ecologists who have field experience with the studied species (hereafter referred to as a group of experts), who defined the classification criteria for the photographic records—the phenophases—characteristics of eachphase, and possible ratings; and (3) a group of 49 citizen scientists (hereafter called volunteers) who were divided into two groups: with and without training, for the subsequent classification of the photographs.

**Fig 1:**
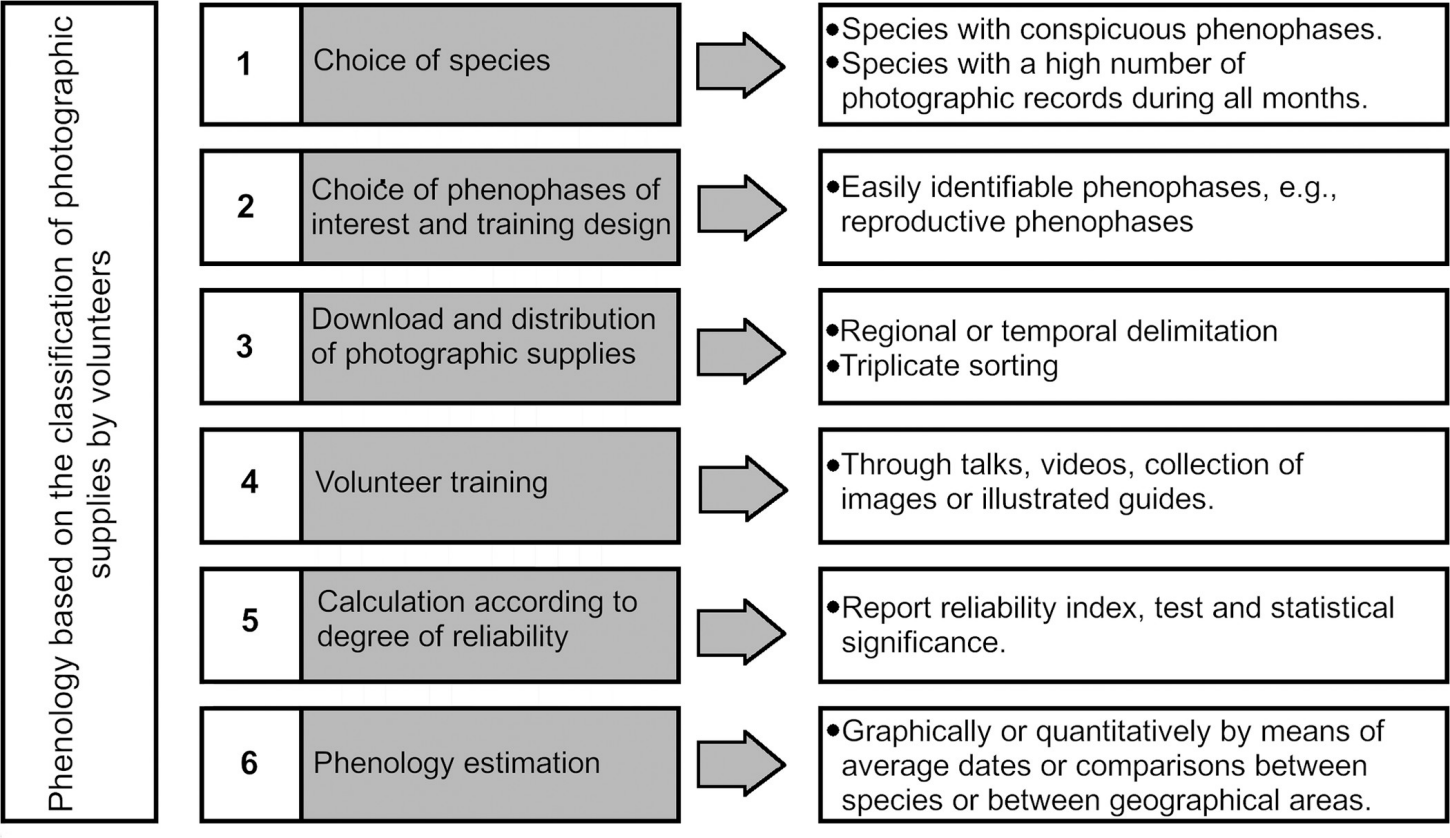
Flowchart followed to generate the phenology of species from records of iNaturalist.

Two species of invasive plants in Mexico were used to develop the proposed methodology and identify the potential difficulties with it (Fig 1). Since the methodology focused on generating phenological data at a regional or larger scale, we select species that have a wide distribution, and a large number of records to evaluate phenological information over one year. Invasive species, although they are not the only species that have these characteristics, nor are they necessarily the species with the highest number of observations in Naturalista (www.naturalista.mx), provide good case studies because, they offer large amounts of data over a large geographical area. The presence of clear phenological phases was an important criterion, so the selected invasive species had conspicuous reproductive and vegetative structures (*i*.*e*., with considerable size as well as a vibrant color and shape easily distinguished from the rest of the plant). Two species that fulfilled the above criteria were selected for the study: *Leonotis nepetifolia* (L.) R. Brown (1,234 records) and *Nicotiana glauca* Graham (1,583 records). For both species, four easily identifiable phenological stages were defined: (1) initial growth, which corresponds to the presence of leaf buds and young leaves identified by their shape, size, and color; (2) immature flower, which corresponds to flower buds; (3) mature flower, which includes flowers with and unfolded corolla; and (4) dry fruits.

Once the phenophases were selected, the group of experts designed an informative guide with the main characteristics of the plant structures that define each phenophase for each species, detailing the specific structures to be identified in each phenophase and the scoring protocol. Not all phenophases and structures were easily identifiable through photographs. For example, for *L*. *nepetifolia*, the immature fruit remains within one of the bracteoles that are grouped in globose verticils; but it can be difficult to distinguish when an apparently empty bracteole corresponds to the early stages of a flower bud and when it corresponds to an immature fruit. For that reason, we suppressed the phenophases "immature fruit" and "mature fruit" and only used "dry fruit", which in both species is easily identifiable by its change in coloration from green to brown.

The illustrated guide was used to train a subgroup of volunteers, hereafter referred to as trained volunteers. The volunteers were asked to classify the photographs, with sessions lasting approximately 30 minutes per species. During each session, the chosen vegetative and reproductive structures were described and the structures to be scored clearly explained. At the end of each session, the group of experts scored a sample of 30 records of the select species with the help of the volunteers. This random sample corresponded to 3% of the total records for each species. Each trained volunteer was provided with the illustrated guide for later consultation and a set of post-training photographs that they classified without assistance to identify possible errors or omissions during the training session. The training session was complemented by an additional session dedicated to questions by volunteers. The subgroup of volunteers without training did not receive any information nor material.

**Fig 2:**
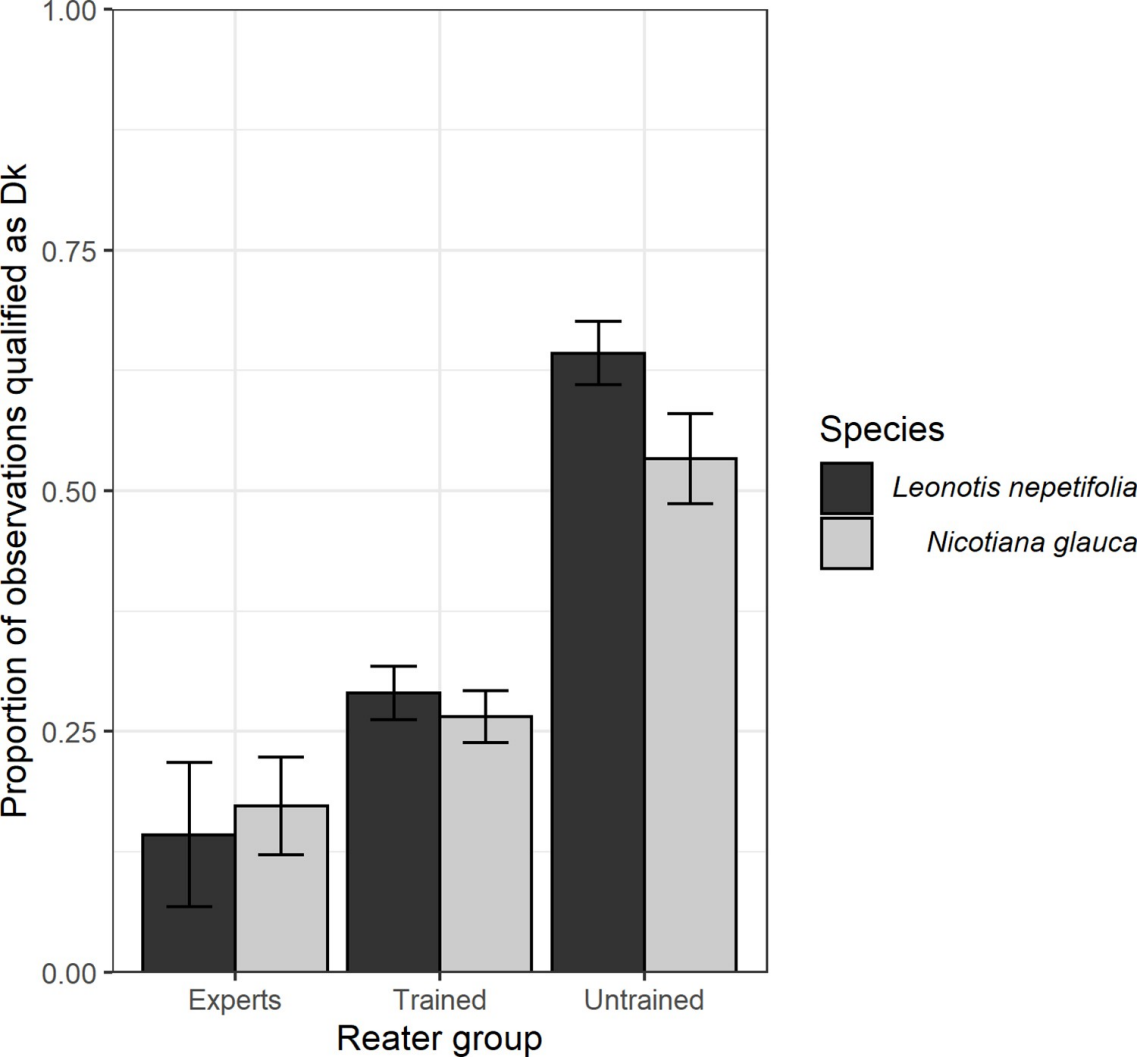
Two views of observations reviewed by volunteers on the Naturalista website. A record of each species used in this study, *Leonotis nepetifolia* (top) and *Nicotiana glauca* (bottom), is shown. The *N*. *glauca* observation is composed of two photographs, marked on the image with a blue box. The photograph of *L*. *nepetifolia* shows the phenophases of new growth, immature flower, mature flower, and dried fruit. The photograph of *N*. *glauca* shows the immature flower, mature flower, and dried fruit phenophases.

On the Naturalista platform, when a user registers an observation, it may consist of one or more photographs with the same georeference and date assigned (Fig 2). For the proposes of this paper, the unit of observation was the record, not the individual photographs. The data with the total number of observations of each species available for Mexico (up to July 19, 2020) were downloaded from the Naturalista website (www.Naturalista.mx), *Leonotis nepetifolia* (1,234 records) and *Nicotiana glauca* (1,583 records), and randomly assigned into 29 subgroups of similar size, hereafter databases.

Each database had a set of approximately 30 to 50 observations. Each observation consisted of a URL link that redirected the rater to the observation on the website (Fig 2) as well as fixed fields that could be scored by the volunteers, and that corresponded to each phenophase. Each observation had date and geographical information as well as information about observer. In addition to the phenophases to be scored, a field defined as "Whole plant" was included to identify whether the observation was made on a whole individual or on a section of an individual. Volunteers were also asked to record the time taken to score each database. Phenophases could be rated with one of three ratings, "Yes” (Y), "No” (N), and "Don’t know" (Dk), but each observation could have more than one phenophase at the same time. "Yes" and "No" ratings correspond to whether the phenophase is distinguishable or not in the observed photographic record while "Don’t know" was used in cases where the observed photograph(s) was unclear, did not correspond to the species of interest, or was clearly a cultivated individual (*e*.*g*., in a pot).

Each volunteer was assigned at least three different databases (30 to 50 records per database), to ensure that each observation was independently reviewed and scored by three trained and three untrained volunteers. In addition, the expert group rated a total of three *L*. *nepetifolia* databases (84 records) and four *N*. *glauca* databases (214 records). The groups of trained and untrained volunteers were different for each species to avoid a possible bias due to learning experience. In total, each rater scored three databases, except for *L*. *nepetifolia* reviewers without training, who were assigned five databases per rater.

### Statistical analysis

The level of agreement was calculated by group of raters (experts, trained volunteers, and untrained volunteers) and by phenophase, for each species, using Fleiss’ Kappa index (K) [42]. This index measures the degree of agreement between *n* raters considering all the categories in which it can be classified, in this case S, N, Dk, and considering that the identity of the raters may be different for different study subjects, in this case, photographic records. The "irr" package version 0.84.1 [43] was used to calculate the index and its statistical significance by means of a two-tailed *z*-test. To estimate whether the level of inter-rater agreement differed between groups, for each phenophase a $x_{equals\;k^{\prime }s}^{2}$, test was performed, which is a test originally proposed to evaluate differences between Kappa indices. If significant differences were found between groups of raters, two-tailed z-tests were performed per rater group pair [44], for which the standard error of the mean obtained by bootstrapping with 1,000 random resamples was used [45]. Additionally, the distributions of the K-index frequencies for each group were analyzed by bootstrapping, and the results were compared to what was found by the $x_{equals\;k^{\prime }s}^{2}$, tests (S1–S4 Figs). Generalized linear models with a binomial distribution and logarithmic link function [46] were used to explore the relationship between the proportion of observations rated as Dk as the dependent variable, and the rating group, the species rated and the interaction between the latter two factors as independent variables. The species factor was considered to determine whether the ease or difficulty of the volunteers in classifying phenophases is independent of the species if species with clear phenophases are considered. The model chosen, following a backward selection, retained only the components that were significant. Subsequently, Tukey tests were performed for multiple contrasts using version 1.4–19 of the “multcomp” package [47]. All computations and data analyses were performed using R 4.0.0 [48], with evaluations of statistical significance made at α = 0.05.

## Results

### Classification of photographic records and comparison between trained and untrained groups of volunteers

The results were based on the classifications made by 24 trained volunteers, 25 untrained volunteers and five experts. Of the trained group, the 24 volunteers classified 1,014 observations of *L*. *nepetifolia* and 23 classified 1,044 observations of *N*. *glauca*. The observations were therefore evaluated independently three times by three groups (nine independent raters per observation). Eleven volunteers from the untrained group classified 811 records of *L*. *nepetifolia* and 14 classified 433 observations of *N*. *glauca*. The five experts participated in classifying 84 observations of *L*. *nepetifolia* and four of them were involved in classifying 214 observations of *N*. *glauca* (S1 Appendix).

**Fig 3:**
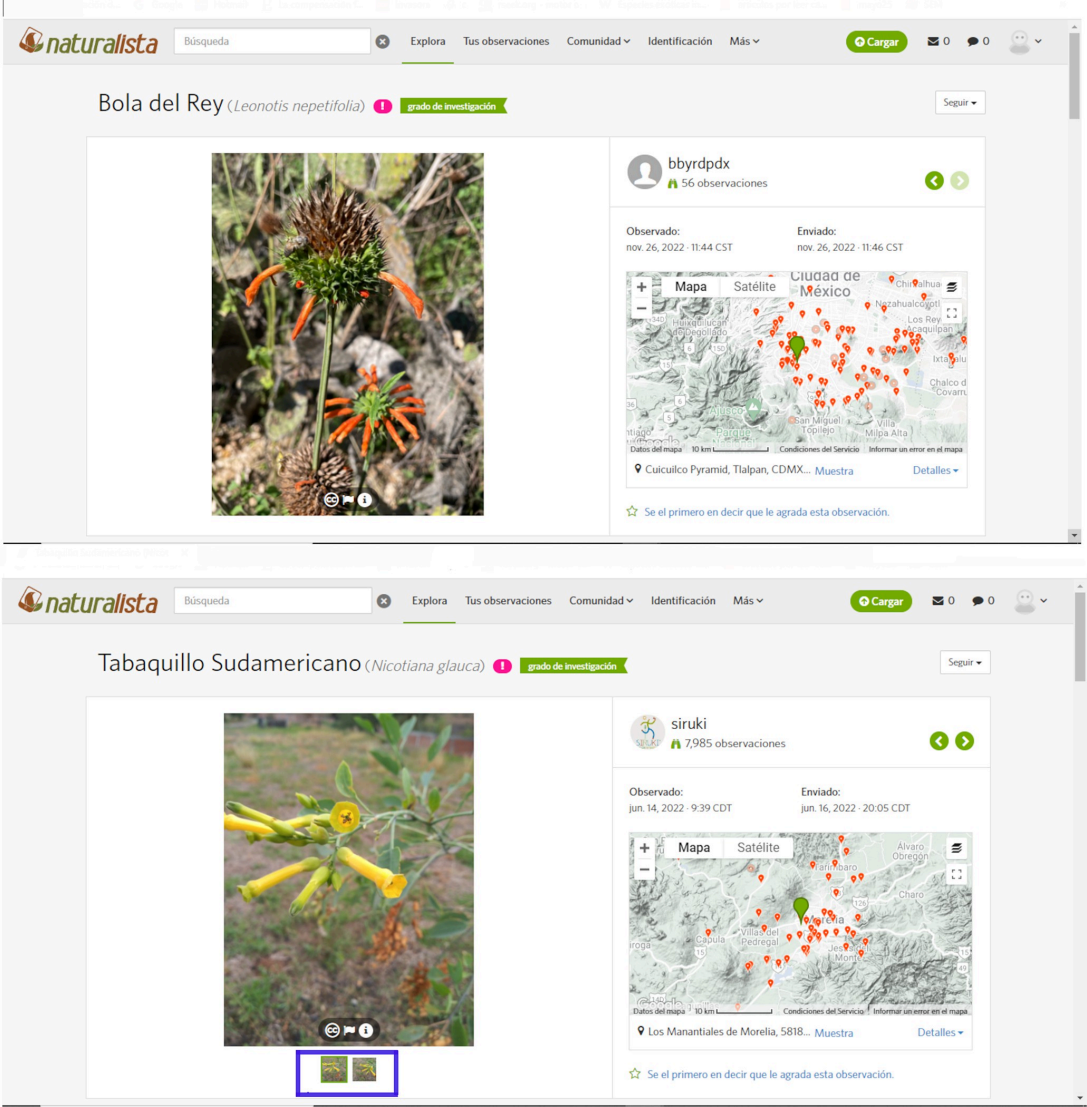
Proportion of records classified as DK (Don’t know) (± CI 95%) for each rater group. According to multiple contrasts by Tukey tests, all rater groups are different.

The proportion of observations scored as Dk varied between rater groups (χ^2^ = 179.408, 1.35, *df* = 3, *p* < 0.001) (S2 Appendix). Neither the species nor the species-group interaction was significant, *i*.*e*., the untrained volunteer group rated photographs as Dk in a higher proportion, and the expert group in lower proportion, regardless of species. Neither species nor the species-group interaction was retained in the generalized linear model chosen to explain the proportion of records scored as Dk. The expert group rated observations as Dk in the lowest proportion (16% of observations, *z* = -11.790, *p* < 0.001), followed by the trained group (28%, *z* = 3.245, *p* < 0.001), while the untrained group had the highest incidence of classifying observations as Dk (60%, *z* = 8.094, *p* < 0.001) (Fig 3).

The degree of agreement in the classification of observations varied by phenophase and rater group. For both species, the untrained groups had a very low rate of agreement in identifying new growth (*L*. *nepetifolia*: *K* = 0.253, *z* = 7.59, *p* < 0.001; *N*. *glauca*: K = -0.293, z = -7.99, *p* < 0.001). In contrast, for the reproductive phenophases (immature flower, mature flower and dry fruit) of *N*. *glauca*, the untrained group had a relatively high level of agreement (immature flower: *K* = 0.674, *z* = 16.6, *p* < 0.001; mature flower: *K* = 0.597, *z* = 14.7, *p* < 0.001; dry fruit: *K* = 0.569, *z* = 14, *p* < 0.001), although not as high as the trained group (immature flower: *K* = 0.782, *z* = 37.5, *p* < 0.001; mature flower: *K* = 0.909, *z* = 43.6, *p* < 0.001, dry fruit: *K* = 0.603, *z* = 28.9, *p* < 0.001) or the expert group (immature flower: *K* = 0.885, *z* = 20, *p* = < 0.001; mature flower: *K* = 0.960, *z* = 21.9, *p* < 0.001, dry fruit: *K* = 0.724, *z* = 16.2, *p* < 0.001). For the reproductive phenophases of *L*. *nepetifolia*, as for the new growth phenophase, the agreement rates reached by the untrained volunteers were low (immature flower: *K* = 0.118, *z* = 0.51, *p* < 0.001; mature flower: *K* = 0.262, *z* = 7.73, *p* < 0.001; and dry fruit: *K* = 0.592, *z* = 17.6, *p* < 0.001).

**Fig 4:**
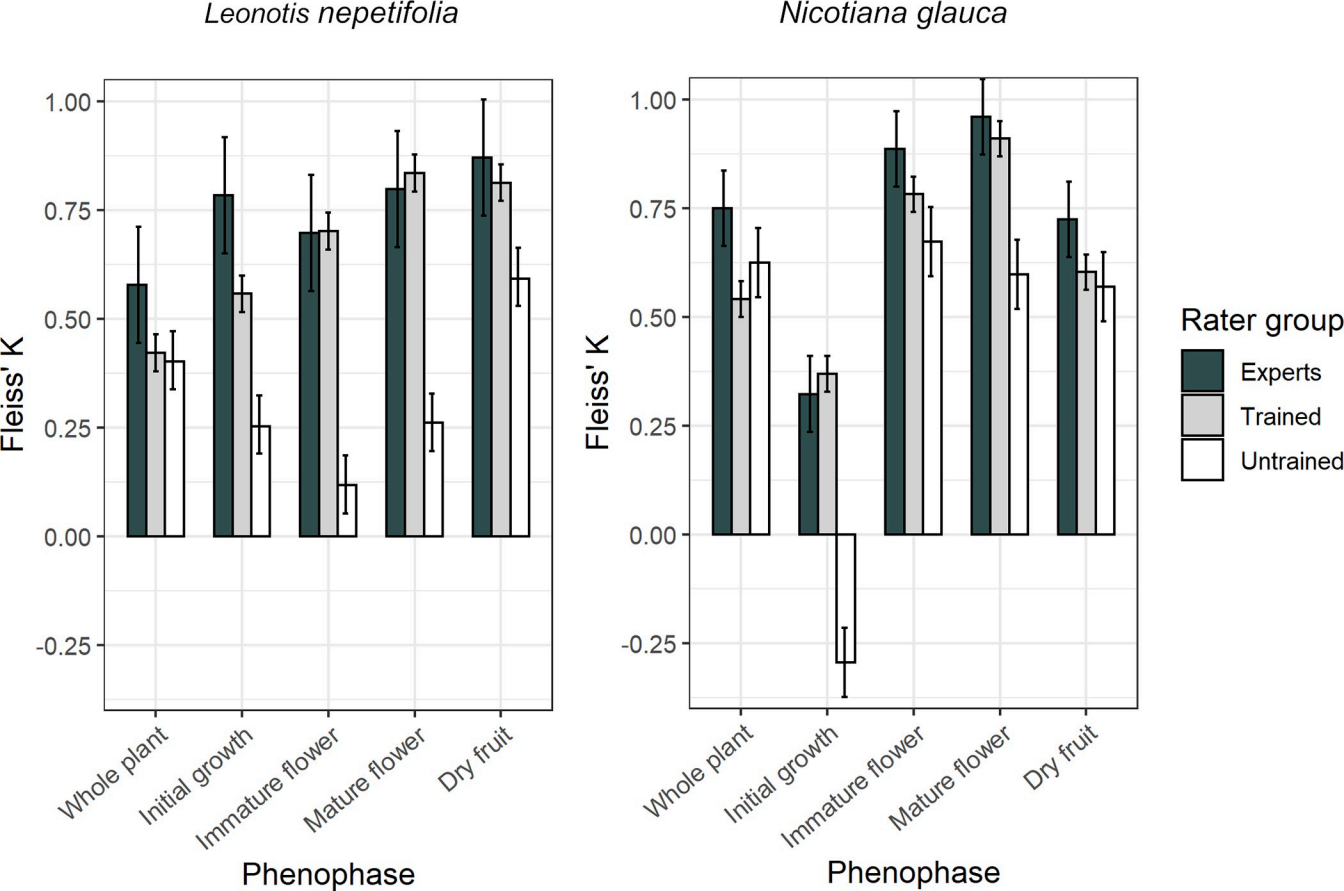
Agreement indices (±CI 95%) for raters in classifying records of *Leonotis nepetifolia* and *Nicotiana glauca*. Fleiss Kappa concordance indices for three groups of raters in classifying photographic records of *L*. *nepetifolia* (left) and *N*. *glauca* (right). Values of K can range from -1 to 1 and represent the proportion of agreement between groups beyond that expected by chance. The index equal to zero represents agreement expected by chance, while values greater than 0.4 are considered good agreement, and greater than 0.75 represent excellent agreement [44,49].

For both species, trained volunteer levels of agreement matched expert levels of agreement for reproductive phenophases (*L*. *nepetifolia* experts, immature flower: *K* = 0.697, *z* = 10.2, *p* < 0.001; mature flower: *K* = 0.798, *z* = 11.7, *p* < 0.001; dry fruit: *K* = 0.870, *z* = 12.8, *p* < 0.001; *L*. *nepetifolia* trained, immature flower: *K* = 0.701, *z* = 32.6, *p* < 0.001; mature flower: *K* = 0.834, *z* = 38.8, *p* <0.001; dry fruit: *K* = 0.813 *z* = 37.8, *p* < 0.001; see above values for *N*. *glauca*), except for the *N*. *glauca* dry fruit phenophase (Fig 4, Table 1). Raters took approximately half a minute to score each observation (experts: $\bar{x}$ = 42±16 seconds; trained: $\bar{x}$ = 45±15; untrained: $\bar{x}$ = 34±14). According to the trained raters, only about 30% of the records for *L*. *nepetifolia* (*K* = 0.46, *z* = 28.5, *p* < 0.001) and *N*. *glauca* (*K* = 0.54, *z* = 26, *p* <0.001) corresponded to whole plants.

**Table 1:** Comparisons and degree of agreement between raters (3 groups) phenophases (three reproductive and two vegetative) and two species (*L*. *nepetifolia* and *N*. *glauca*).

Comparison	*Leonotis nepetifolia*			*Nicotiana glauca*		
	Percentage difference	z-score	$x_{equal\;k^{\prime }s}^{2}$,	Percentage difference	*z*-score	$x_{equal\;k^{\prime }s}^{2}$,
**Immature flower**	** **					
Experts *vs* trained	0.42	-1.07	215.60***	10.42	2.13*	12.55*
Experts *vs* untrained	57.89	137.28***		21.24	3.53*	
Trained *vs* untrained	58.32	336.38***		10.82	2.36*	
**Mature flower**		** **	** **		** **	
Experts *vs* trained	3.69	-0.52	206.55***	5.04	1.03	52.46***
Experts *vs* untrained	53.56	7.05***		34.24	6.02***	
Trained *vs* untrained	57.25	14.26***			6.83***	31.2
**Dry fruit**		** **	** **		** **	
Experts *vs* trained	5.72	0.8	31.86***	12.11	2.47*	7.63*
Experts *vs* untrained	27.77	3.60**		15.48	2.57*	
Trained *vs* untrained	22.04	5.25***		3.38	0.74	
**Initial growth**		** **	** **		** **	
Experts *vs* trained	22.64	3.172*	75.58***	4.61	-0.94	212.54***
Experts *vs* untrained	53.03	6.90***		61.61	10.24***	
Trained *vs* untrained	30.4	7.27***		66.21	14.50***	
**Whole plant**		** **	** **		** **	
Experts *vs* trained	15.61	2.19*	5.41	20.85	4.25***	21.05***
Experts *vs* untrained	17.64	2.29*		12.45	2.07*	
Trained *vs* untrained	2.04	0.49		8.4	-1.84	

All comparisons used Fleiss’ K for raters (expert, trained, and untrained), phenophase and species. Differences between the three rater groups within a phenophase, $x_{equal\;k^{\prime }s}^{2}$ was used for each phenophase, and differences between pairs of rater groups were tested with *z*-tests

*: < 0.05

**: < 0.001

***: < 0.0001.

## Discussion

The reliability of volunteers in citizen science databases has been debated in many studies [50], and often the validity of the observation will depend on the complexity of the observed phenomena [51,52]. In this study, groups of experts and trained raters showed high reliability in classifying reproductive phenophases, indicated by high agreement among raters. However, the classification of less obvious phenophases, such as early growth, which is observed as less conspicuous and contrasting green shoots than flowers, may require greater detail and/or training time [23]. The level of agreement was significantly higher for reproductive phenophases among trained volunteers, compared to untrained volunteers, and for most phenophases the agreement rate among trained volunteers was equal to that of experts. This suggests that phenological assessments from photographs conducted by trained volunteers are as reliable as those performed by expert scientists.

Previous studies have shown that citizen scientists can collect high quality field observations, good enough to be used in original analyses contributing to scientific knowledge generation [23,53–55]. Our results indicate that reliable phenological data can also be generated from photographic sources. Although not all the records reviewed by the volunteers were reviewed by the reviewers, a situation that entails a potential weakness, we believe that this could be partially corrected due to the large number of reviewers, the large number of observations, and the random distribution of the observations that we have. Similar results have been found in other platforms [31,56] such as CrowdCurio, a crowdsourcing-type image annotation tool that involves the participation of financially rewarded, but not necessarily self-interested, non-expert workers who classify photographs for the purposes of some particular project [56]. The use of CrowdCurio also had comparable results between expert and non-experts when classifying photographic inputs for the identification of phenophases in digitized images from herbarium records.

The Naturalista platform has the means to upload non mandatory phenological data at the time of recording an observation, but only about 30% of the photographs of both species reviewed here had a phenological annotation (data from www.naturalista.mx). The high proportion of observations classified as uncertain (Dk) in the group of untrained volunteers suggests that this may be due to the difficulties faced by citizen scientists without training in phenology and plant structures when identifying phenophases, rather than lack of interest, since previous papers have reported that intrinsic motivations, such as interest to generate and share knowledge, are the main motivations for the participation of citizen scientists [57]. Feedback training processes, such as the two-way conversations used in this research [58,59], help citizen science volunteers generate quality information, and professional scientists learned from the needs of the raters to expand the strategies for proper communication between specialized an unspecialized audiences [60].

Our analysis of highlights the benefits of this approach and provides more evidence to support the use of a promising alternative to the limitations posed by specific systems to track phenological data, while also reducing the time and effort required to obtain data [61]. Since this approach does not allow volunteers to corroborate the presence/absence of a particular phenophase, as would happen when rating a live individual, it is paramount to have high-quality images in which the phenophases can be clearly distinguished. Ideally, increased accuracy would be obtained from a series of high- resolution images depicting several portions of the individual, especially with species that have easily identifiable phenophases such as colorful shaped reproductive structures. The application of this methodology to species with less visible structures, as is the case of many grasses whose phenological study is equally important, would be cumbersome to the untrained eye, and would require longer and more detailed training. Furthermore, we want to highlight the fact that photographs of species with inconspicuous structures may become impossible to classify with poor-resolution images, by people without extensive and detailed knowledge about the species, which is the main limitation of using this method followed for all phenophases.

In addition to the obvious taxonomic bias that this entails, it is important to consider other biases into which one could fall, which are also typical of scientific collections. These include biases in taxonomic (such as dominant species in landscapes or preference for certain taxa), morphological (biases towards conspicuous individuals, etc.), geographic [62–64], and temporal preferences [65]. On the other hand, the use of photographs from biodiversity observation platforms presents important advantages. These photographs are a verification tool that phenology platforms do not normally provide, and their opportunistic nature gives observers ease and freedom in data capture, leading to a huge amount of data with geographic and taxonomic coverage that can exceed those of the phenological monitoring platforms [41]. In addition, the presence of photographs makes it easier for researchers to corroborate the identity of the species if they consider it necessary [24,41]. The photographs also allow researchers to carry out a validation when the information obtained is doubtful, for example, when phenological events are identified outside the expected dates or in geographic regions that do not correspond to the known distribution area of the species, or when volunteers have doubts when making a phenological annotation. The Dk category in the classification of phenophases that was followed in this study was used to indicate photographs or phenophases that were blurred, that did not correspond to the species, or that showed evidence of being cultured. However, this scoring can also allow experts to identify if a photograph requires extra peer validation, if phenophase represents a particular difficulty in scoring, or if a more detailed training session is necessary. This category also allows doubtful data to be excluded automatically in a subsequent phenological analysis.

Two of key components of monitoring phenology monitoring are the phenological state and the intensity of the phenophase. The phenological state, defined as presence or absence of a phenophase, provides information on its onset and duration, while the intensity provides information on the temporal distribution of the structures that characterize it [35]. We do not recommend using the method proposed here to calculate population-level phenophase intensity measures, such as detailed phenological peaks, as there may be biases in the availability of photographs that do not allow analysis of phenology at fine temporal resolution [66], as well as limitations in the reliability of volunteers when performing more complex measurements, such as abundance or frequency of species or structures [51,52]. In addition, the low proportion of available observations with whole plants could generate an erroneous estimate of the start or end date of the phenophases, since structures present in a given individual have not been photographed. These incomplete records could be generate false negatives when they report the absence of structures [41]. Our experience suggests that these types of records, for example, incomplete plants without reproductive structures, are a small proportion of total observations, since collectors, expert or not, tend to focus on the most conspicuous structures, such as fruits, flowers, or buds [67–69]. We suggest that the methodology proposed here would be helpful when used to obtain species-level phenological estimates, including mean dates of each phenophase, or comparisons between the temporal distributions of phenophases between species or different geographic areas [51,52].

Ideally, phenological annotations should be conducted by citizen scientists at the time of recording their photographic observation. However, due to the large amount of information without phenological annotations to date, *a posteriori* classification of photographs was performed (such as the one in this study carried out on photographs taken by citizen scientists), can help expand the use of primary data. Approaching citizen science with a big data approach would entail taking advantage of emerging data that have not been generated under the target of interest, but can nonetheless be useful in phenological or climate change studies [70]. Our study found that this approach must be underpinned by training provided to volunteers or citizen scientists assessing phenological data in order to have reliable data. Several training tools can be used to increase the levels of accuracy as part of the training protocols, including informative talks, interactive forums, short videos, image collections or illustrated guides.

The large amounts of phenological information generated through this approach can also be matched against herbarium records [67], and would represent, over time, a temporal continuum of data on the presence of species and the corresponding phenology. Finally, the applicability of data generated through this approach ranges from use in environmental suitability analyses [37–40], documenting large-scale phenological changes (such as in the phenological response of invasive species to new environments and climates) [6], possible changes in the nutrient cycles of invaded ecosystems due to changes in leaf phenology [7], and the phenological response of plant species to GCC [12].

## Supporting information

S1 Fig Frequency distributions of Fleiss’ K values of the three groups of raters, for the immature flower phenophase. Dark gray: Expert group, light gray: Trained volunteers, white: Untrained volunteers Data from *Leonotis nepetifolia* (left) and *Nicotiana glauca* (right).
(TIF)

S2 Fig Frequency distributions of Fleiss’ K values of the three groups of raters for the mature flower phenophase. Dark gray: Expert group, light gray: Trained volunteers, white: Untrained volunteers Data from *Leonotis nepetifolia* (left) and *Nicotiana glauca* (right).
(TIF)

S3 Fig Frequency distributions of Fleiss’ K values of the three groups of qualifiers for the dry fruit phenophase. Dark gray: Expert group, light gray: Trained volunteers, white: Untrained volunteers. Data from *Leonotis nepetifolia* (left) and *Nicotiana glauca* (right).
(TIF)

S4 Fig Frequency distributions of Fleiss’ K values of the three groups of raters for the initial growth phenophase. Dark gray: Expert group, light gray: Trained volunteers, white: Untrained volunteers. Data from *Leonotis nepetifolia* (left) and *Nicotiana glauca* (right).
(TIF)

S1 Appendix Records rated by each rating group. The different spreadsheets show the reviewed observations of each species, with the classifications made by each rating group.
(XLSX)

S2 Appendix Results of the generalized linear model to test the relationship between observations rated as Dk and rating group. Generalized linear models with a binomial distribution and logarithmic link function used to explore the relationship between the proportion of observations rated as Dk (Don’t know) as the dependent variable, and the rating group as independent variables.
(DOCX)
