# Supplementary figures and images for: Practice makes the expert: The importance of training volunteers in the generation of phenological data from photographs of biodiversity observation platforms
Source: PLoS One. 2023 Mar 7;18(3):e0282750. doi: 10.1371/journal.pone.0282750 (PMC9990930; doi:10.1371/journal.pone.0282750)

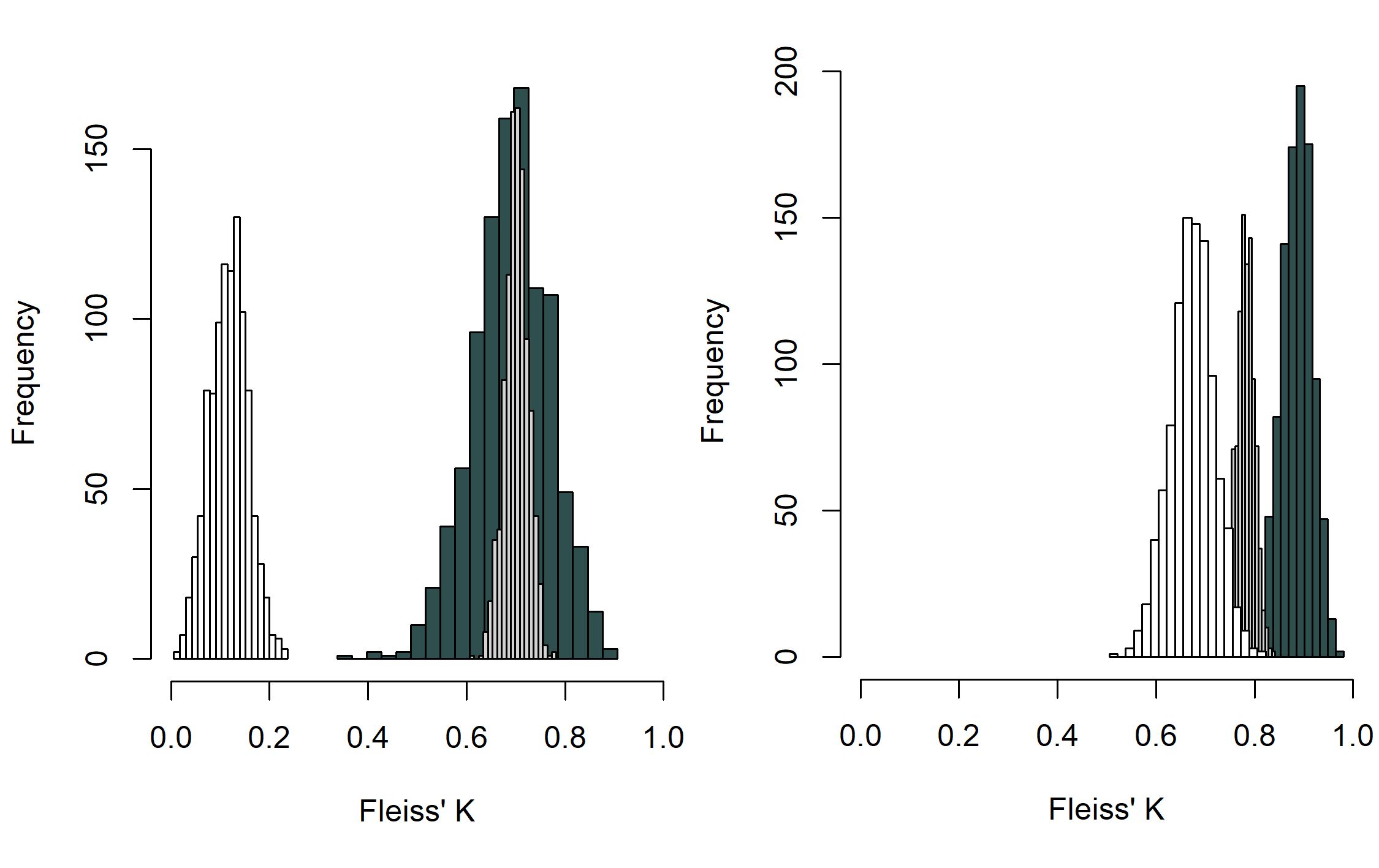

Supplement: S1 Fig — Dark gray: Expert group, light gray: Trained volunteers, white: Untrained volunteers Data from Leonotis nepetifolia (left) and Nicotiana glauca (right). (TIF) [file pone.0282750.s001.tif]

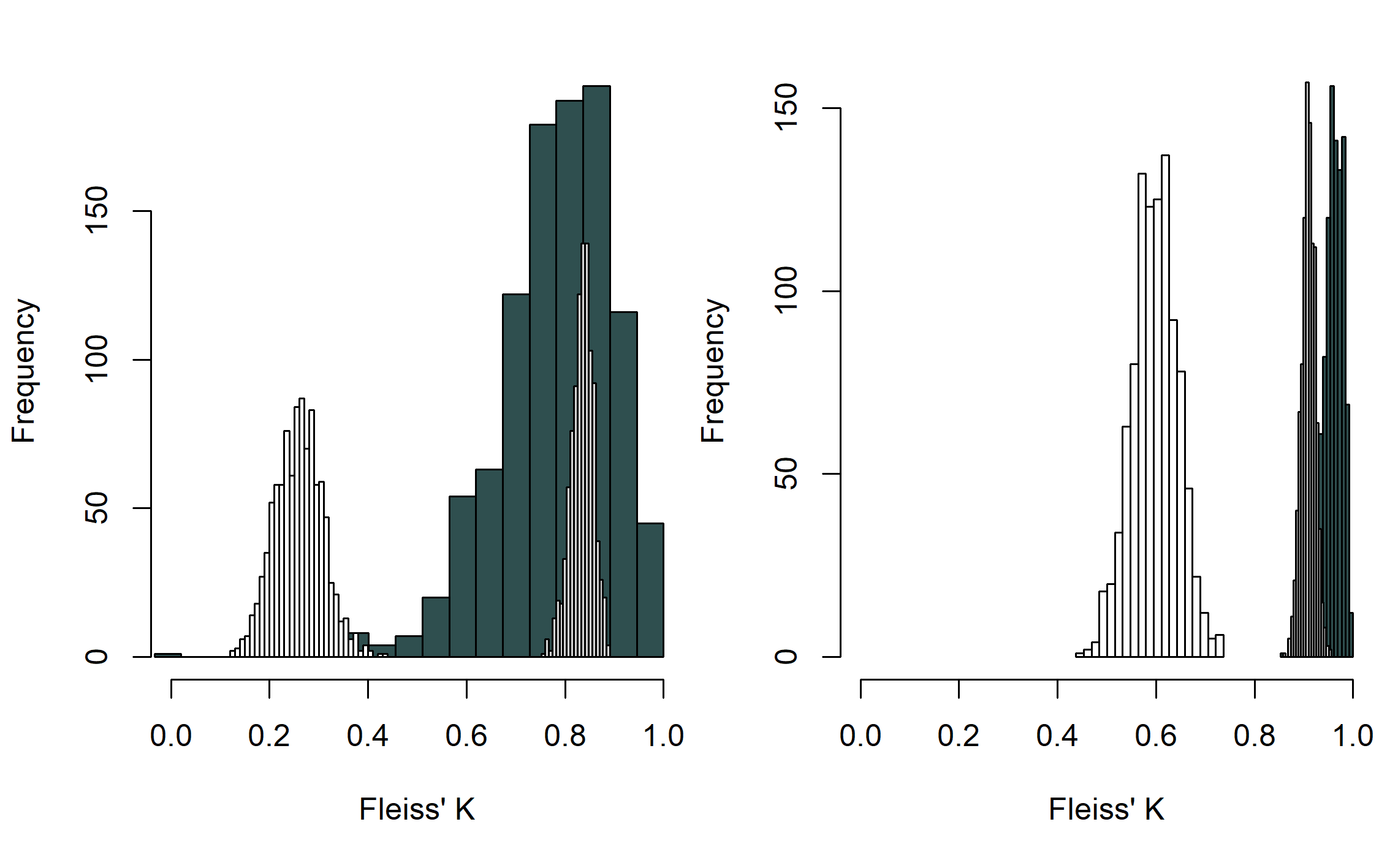

Supplement: S2 Fig — Dark gray: Expert group, light gray: Trained volunteers, white: Untrained volunteers Data from Leonotis nepetifolia (left) and Nicotiana glauca (right). (TIF) [file pone.0282750.s002.tif]

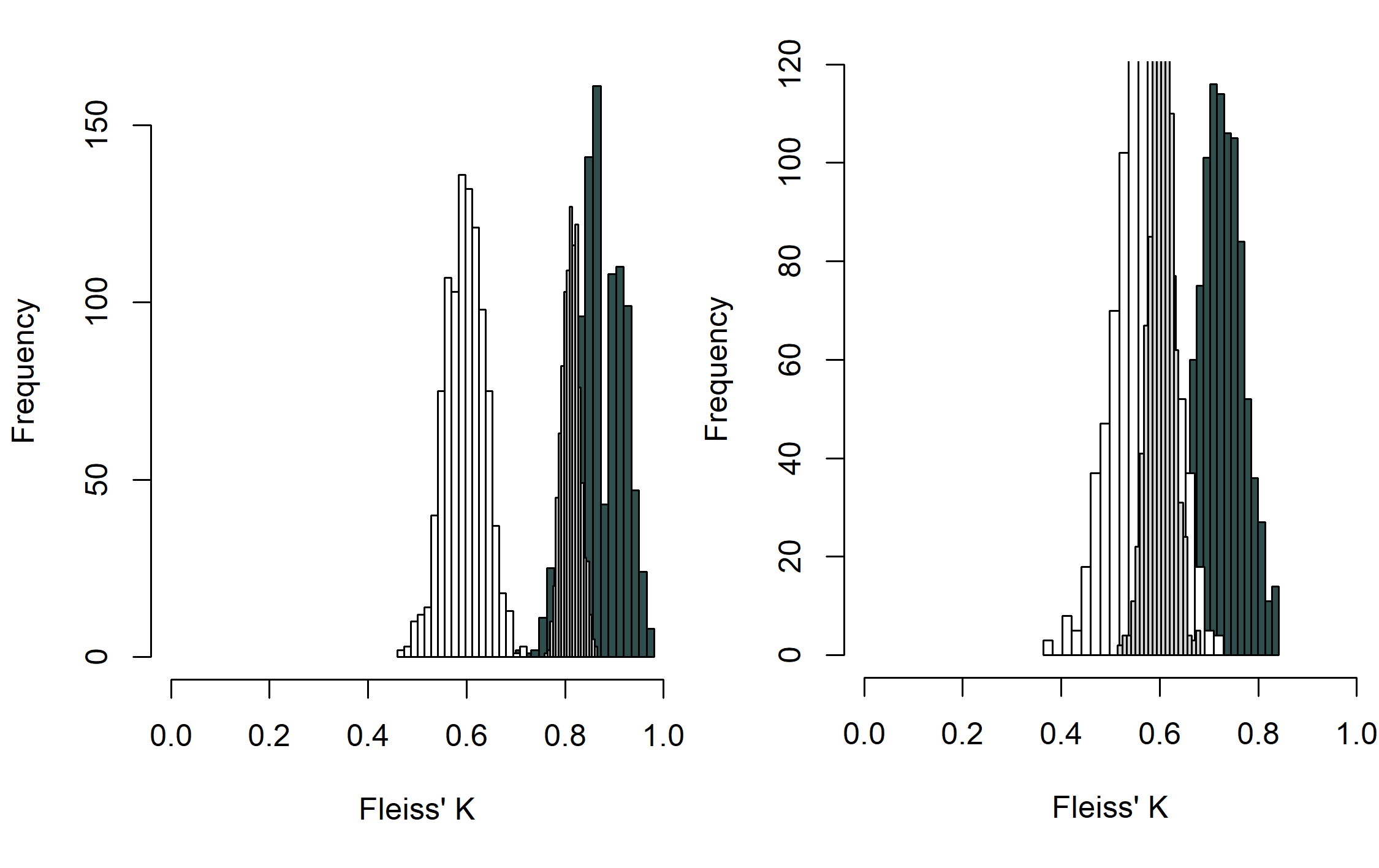

Supplement: S3 Fig — Dark gray: Expert group, light gray: Trained volunteers, white: Untrained volunteers. Data from Leonotis nepetifolia (left) and Nicotiana glauca (right). (TIF) [file pone.0282750.s003.tif]

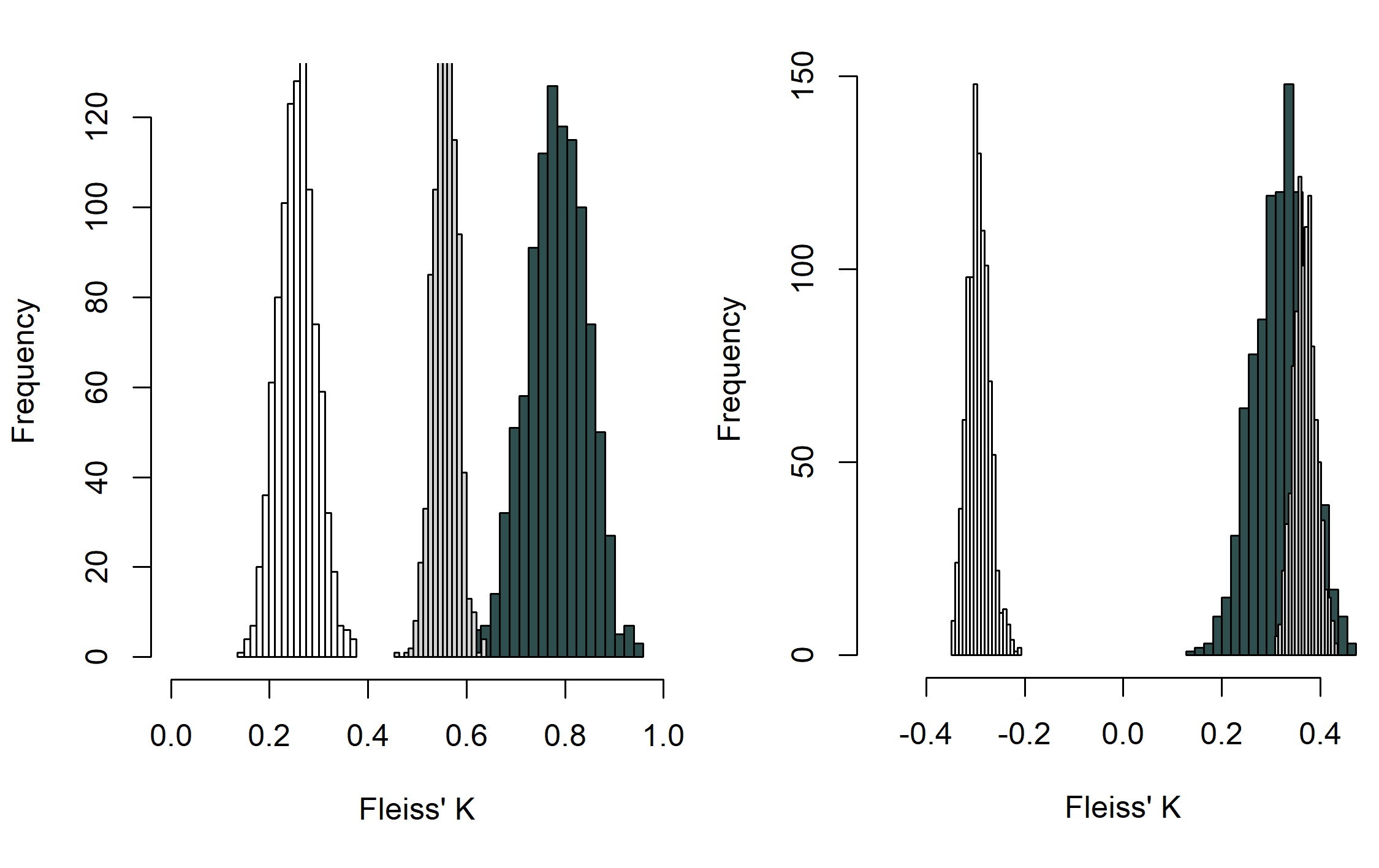

Supplement: S4 Fig — Dark gray: Expert group, light gray: Trained volunteers, white: Untrained volunteers. Data from Leonotis nepetifolia (left) and Nicotiana glauca (right). (TIF) [file pone.0282750.s004.tif]
