# Supplementary material for: Practice makes the expert: The importance of training volunteers in the generation of phenological data from photographs of biodiversity observation platforms
Source: PLoS One. 2023 Mar 7;18(3):e0282750. doi: 10.1371/journal.pone.0282750 (PMC9990930; doi:10.1371/journal.pone.0282750)
Supplement: S2 Appendix — Generalized linear models with a binomial distribution and logarithmic link function used to explore the relationship between the proportion of observations rated as Dk (Don’t know) as the dependent variable, and the rating group as independent variables. (DOCX) [file pone.0282750.s006.docx]

**S1 Appendix.** **Results of the generalized linear model to test the relationship between observations rated as Dk and rating group.** Generalized linear models with a binomial distribution and logarithmic link function used to explore the relationship between the proportion of observations rated as Dk as the dependent variable, and the rating group as independent variables.

glm(formula = yvar ~ gpo, family = binomial, data = tablNS)

Pseudo R^2^ McFaden: 0.806

Model equation: $p=\frac{1}{1+e^{-(-1.80+0.52x+1.30x)}}$

|  | **Coefficients** | **Std. Error** | ***z* value** | ***p*-value** |
| --- | --- | --- | --- | --- |
| **Intercept** | -1.8053 | 0.1542 | -11.711 | <0.001 |
| **Group trained** | 0.5232 | 0.1612 | 3.245 | 0.0011 |
| **Group untrained** | 1.3024 | 0.1609 | 8.094 | <0.001 |

(Dispersion parameter for binomial family taken to be 1)

Null deviance: 184.159 on 5 degrees of freedom

Residual deviance: 4.751 on 3 degrees of freedom

AIC: 49.196

Number of Fisher Scoring iterations: 3
